# Artificial neural network identified a 20‐gene panel in predicting immunotherapy response and survival benefits after anti‐PD1/PD‐L1 treatment in glioblastoma patients

**DOI:** 10.1002/cam4.7218

**Published:** 2024-05-11

**Authors:** Yaning Wang, Zihao Wang, Xiaopeng Guo, Yaning Cao, Hao Xing, Yuekun Wang, Bing Xing, Yu Wang, Yong Yao, Wenbin Ma

**Affiliations:** ^1^ Department of Neurosurgery, Center for Malignant Brain Tumors, National Glioma MDT Alliance, Peking Union Medical College Hospital Chinese Academy of Medical Sciences and Peking UnionMedical College Beijing China

**Keywords:** artificial neural network model, glioblastoma, immune checkpoint inhibitor, immunotherapy

## Abstract

**Background:**

Immune checkpoint inhibitors (ICIs) are a promising immunotherapy approach, but glioblastoma clinical trials have not yielded satisfactory results.

**Objective:**

To screen glioblastoma patients who may benefit from immunotherapy.

**Methods:**

Eighty‐one patients receiving anti‐PD1/PD‐L1 treatment from a large‐scale clinical trial and 364 patients without immunotherapy from The Cancer Genome Atlas (TCGA) were included. Patients in the ICI‐treated cohort were divided into responders and nonresponders according to overall survival (OS), and the most critical responder‐relevant features were screened using random forest (RF). We constructed an artificial neural network (ANN) model and verified its predictive value with immunotherapy response and OS.

**Results:**

We defined two groups of ICI‐treated glioblastoma patients with large differences in survival benefits as nonresponders (OS ≤6 months, *n* = 18) and responders (OS ≥17 months, *n* = 8). No differentially mutated genes were observed between responders and nonresponders. We performed RF analysis to select the most critical responder‐relevant features and developed an ANN with 20 input variables, five hidden neurons and one output neuron. Receiver operating characteristic analysis and the DeLong test demonstrated that the ANN had the best performance in predicting responders, with an AUC of 0.97. Survival analysis indicated that ANN‐predicted responders had significantly better OS rates than nonresponders.

**Conclusion:**

The 20‐gene panel developed by the ANN could be a promising biomarker for predicting immunotherapy response and prognostic benefits in ICI‐treated GBM patients and may guide oncologists to accurately select potential responders for the preferential use of ICIs.

## 1 INTRODUCTION

Glioblastoma is the most common and lethal primary brain tumor exhibiting high aggressiveness, with a 5‐year overall survival (OS) rate of approximately 10% and a median OS time of 14–16 months.^1^ Currently, the standard treatment for glioblastoma includes maximal surgical resection, concurrent chemoradiotherapy, and adjuvant temozolomide (TMZ) chemotherapy. In recent years, tumor‐treating fields (TTF) have also been recommended as one of the treatment options for glioblastoma as they can significantly prolong the survival of patients.^2^ Targeted therapy and immunotherapy have also been extensively studied in glioblastoma, but neither of them has been found to prolong the OS of unselected patients. Therefore, glioblastoma remains the greatest therapeutic challenge with extremely limited treatment options.

Among all types of immunotherapy, immune checkpoint inhibitors (ICIs) have shown the greatest efficacy in the treatment of multiple cancers.^3^ However, for glioblastoma, anti‐PD‐1/PD‐L1 immunotherapy has shown limited satisfactory results in clinical trials, revealing low tumor response and no prolongation of patient survival, which limits its therapeutic application in glioblastoma.^4^ Previous studies reported that tumor mutation burden (TMB), neoantigen load, mismatch repair, checkpoint molecule expression, immune cell infiltration/exclusion, and T‐cell receptor clonality might correlate with clinical benefit across multiple cancers.^5^, ^6^ However, the predictive effect of these biomarkers on glioblastoma immunotherapy is not clear, and further studies are needed to verify it.

Artificial neural network (ANN), a form of machine learning, is an electronic analog of the biological nervous system. It can be trained to identify data in complex patterns.^7^ ANN has been widely applied to cancer studies,^8^, ^9^, ^10^ mostly in the diagnosis and grading of the disease. However, a lack of research on the prediction of the immunotherapy effect of cancer patients using ANN remains.

Considering the poor prognosis of glioblastoma patients and the lack of predictive biomarkers for immunotherapy, we analyzed the data of 81 glioblastoma patients treated with ICIs, aiming to explore potential indicators for predicting the immunotherapy response and prognostic benefits of ICIs. The results of this research may be able to guide oncologists in selecting potential immunotherapy responders for the preferential use of ICIs.

## 2 MATERIALS AND METHODS

### 2.1 Patient population and data acquisition

A total of 83 glioblastoma patients, confirmed by pathology, underwent at least one dose of anti‐PD1/PD‐L1 immunotherapy (nivolumab, pembrolizumab, atezolizumab, avelumab, or durvalumab) from a large‐scale clinical trial (ICI‐treated cohort). They underwent genomic profiling using the FDA authorized Integrated Mutation Profiling of Actionable Cancer Targets (MSK‐IMPACT) assay for testing somatic mutations in a subset of 468 cancer‐related genes using both tumor‐derived and matched germline normal DNA after ICI administration.^11^ The somatic mutational and clinical data of the ICI‐treated cohort were downloaded from cBioPortal (https://www.cbioportal.org/study/summary?id=tmb_mskcc_2018), and two patients were excluded due to the lack of follow‐up time (Table S1).^12^ In the ICI‐treated cohort, OS was defined from the date of the first ICI administration to the time of death or the last follow‐up. Additionally, somatic mutation data based on the whole‐exome sequencing platform, level three RNA sequencing data based on the Illumina HiSeq platform, and corresponding clinical information of The Cancer Genome Atlas (TCGA) glioblastoma cohort with no ICI treatment were also downloaded (https://portal.gdc.cancer.gov/), and 364 patients who possessed the above data simultaneously were enrolled (Table S1). Informed consent was obtained from all participants in the ICI‐treated cohort and TCGA cohort.

### 2.2 Identification of responders and nonresponders to immunotherapy

We defined the clinical response to immunotherapy by the quartiles of OS time. First, the ICI‐treated patients were ranked by their OS times in ascending order. Among the first quartile of patients, those who died with OS ≤6 months were defined as nonresponders, and among the fourth quartile of patients, those who survived more than 1 year at the last follow‐up were defined as responders. The mutation types and frequencies of genes were visualized and compared between responders and nonresponders by using the maftools package in R software.^13^ Several previously reported predictors of prognosis and response to ICI treatment in multiple cancers were also compared between the two groups. Nonsynonymous somatic TMB and tumor purity were measured by MSK‐IMPACT. Mutant‐allele tumor heterogeneity (MATH), a novel measure of intratumor genetic heterogeneity, was assessed by the maftools package.^14^ The neoantigen counts and clonality of mutations were characterized as described by Charoentong et al.^15^ The copy number alteration (CNA) burden was defined as the total number of genes with copy number amplifications or deletions in each sample.^16^

### 2.3 Machine learning algorithms for predicting responders

First, random forest (RF) was performed on the training set, composed of previously described responders and nonresponders, to select the most critical responder‐relevant features by calculating the importance score for each variable via the randomForest package in R software. The mutation status of all genes was chosen as the input factor (independent variable), and the status of immunotherapy response (responder as 1 and nonresponder as 0) was chosen as the outcome (binary dependent variable). Ten‐fold cross‐validation was performed to determine the optimal number of variables with low cross‐validation error. Then, those variables were selected as the input layer of the artificial neural network (ANN) model, which was developed by using the neuralnet package. We trained a feed‐forward ANN with five sigmoid hidden neurons (nodes) and one output neuron, and a scaled conjugate gradient back‐propagation algorithm was also applied to adjust weighted values on the dedicated partition.^17^ The predictive ability of the ANN was evaluated by the area under the receiver operating characteristic (ROC) curve (AUC), sensitivity, specificity, negative predictive value (NPV), positive predictive value (PPV), accuracy, precision, and F1 score.^18^ The performance metrics of different models were compared by the DeLong test.

Subsequently, the ANN model constructed by the training set was applied to the total ICI‐treated patients and TCGA patients to predict potential nonresponders and responders to immunotherapy. Kaplan–Meier survival analysis was then performed to evaluate the OS of responders and nonresponders, and survival differences were assessed by a two‐sided log‐rank test. Tumor Immune Dysfunction and Exclusion (TIDE) is a known algorithm for predicting the clinical response to immunotherapy based on transcriptomic profiles of tumor samples.^19^ A TIDE score <0 was considered to be sensitive to immunotherapy, and >0 to be resistant to immunotherapy. The TIDE scores of responders and nonresponders were compared by the Mann–Whitney *U* test. The statistical analyses in this study were performed by using R software (version 3.6.1). A two‐tailed *p* value <0.05 was considered statistically significant.

## 3 RESULTS

### 3.1 Comparisons of genomic variations between responders and nonresponders

**FIGURE 1:**
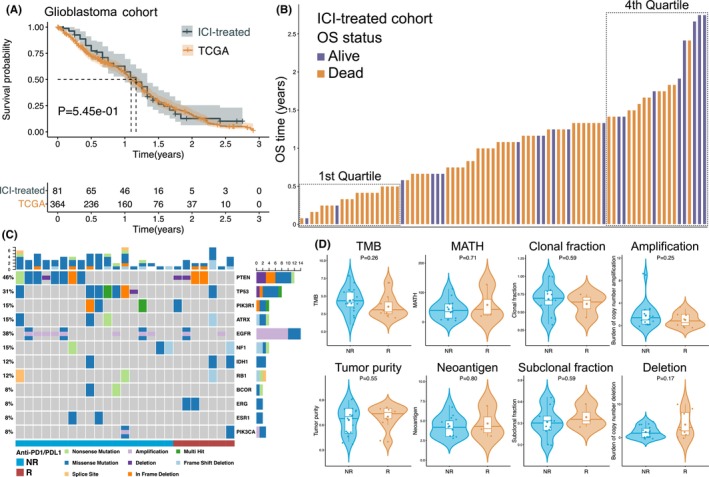
Comparisons of genomic variations between responders and nonresponders of glioblastoma patients. (A) Kaplan–Meier survival analysis of the OS rate in two glioblastoma cohorts, including the ICI‐treated cohort and TCGA cohort without immunotherapy. (B) Bar plot demonstrating the ICI‐treated patients ranked by OS time in ascending order. There were 18 nonresponders with OS ≤6 months in the 1st quartile and eight responders with OS ≥17 months in the 4th quartile. (C) Differential genomic variation analysis of the 12 top mutated genes (mutation frequencies >5%) between responders and nonresponders. The bar plots in the upper and right panels represent counts of somatic mutations and copy number alterations. (D) Comparisons of several previously reported predictors of prognosis and response to ICI treatment between responders and nonresponders. NR is short for nonresponder, and R is short for responder.

Survival analysis demonstrated no significant difference in the OS rate between the ICI‐treated (receiving anti‐PD1/PD‐L1 immunotherapy) and TCGA (without ICI treatment) glioblastoma patients (*p* = 0.55) (Figure 1A). We speculated that this may be because most glioblastoma patients in the ICI‐treated cohort did not respond to ICI treatment. Hence, we need to identify the specific glioblastoma patients who would respond to immunotherapy and indicators for predicting responders. According to the criteria, there were 18 nonresponders with OS ≤6 months and eight responders with OS ≥17 months, which constituted the training set (Figure 1B). The waterfall plot with the top mutated genes (mutation frequencies >5%) is displayed in Figure 1C, and there was no significant difference in the mutation frequency of each gene between two groups. In addition, several previously reported predictors of prognosis and the immunotherapy response in multiple cancers, such as TMB, did not differ significantly between responders and nonresponders in glioblastoma (Figure 1D).

Regarding the clinical features, we found that patients' age and MGMT promoter methylation status had no significant impact on the efficacy of immunotherapy in either the ICI‐treated cohort or the TCGA cohort. In addition, there was no significant difference in the efficacy of immunotherapy in primary glioblastoma and recurrent glioblastoma in the two cohorts, which is consistent with previous phase III clinical trials: no matter the clinical trials of CHECKMATE 143 in recurrent glioblastoma or CHECKMATE 498 and CHECKMATE 548 in primary glioblastoma, ICI treatment could not effectively prolong the survival of patients.^4^, ^20^ We also found that among ICI‐treated cohort, the proportion of males was significantly higher in responders (*p* = 0.024), but this conclusion has not been verified in TCGA samples. Therefore, whether it has clinical significance needs further research in the future. Meanwhile, a higher proportion of responders have IDH mutations (*p* < 0.001), which, to a certain extent, also explains the important role of this gene mutation in immunotherapy (Table 1).

**TABLE 1:** Comparisons of the demographic and clinicopathological features of responders and nonresponders to anti‐PD1/PD‐L1 therapy.

Characteristic	No. (%)								
	ICI‐treated training cohort (*n* = 26)			ICI‐treated total cohort (*n* = 81)			TCGA cohort (*n* = 364)		
	NR (*n* = 18)	R (*n* = 8)	*p* Value	ANN‐predicted NR (*n* = 62)	ANN‐predicted R (*n* = 19)	*p* Value	ANN‐predicted NR (*n* = 308)	ANN‐predicted R (*n* = 56)	*p* Value
Age, median (range), years	54.5 (15–77)	55.5 (29–67)	0.806	53.0 (15–77)	50.0 (22–80)	0.234	62.0 (23–89)	60.0 (21–82)	0.322
Sex									
Male	9 (50.0)	8 (100)	**0.023**	37 (59.7)	17 (89.5)	**0.024**	196 (63.6)	37 (66.1)	0.727
Female	9 (50.0)	0 (0)		25 (40.3)	2 (10.5)		112 (36.4)	19 (33.9)	
MGMT promoter status									
Methylated	8 (44.4)	5 (62.5)	0.673	19 (30.6)	8 (42.1)	0.354	130 (42.2)	25 (44.6)	0.735
Unmethylated	10 (55.6)	3 (37.5)		43 (69.4)	11 (57.9)		178 (57.8)	31 (55.4)	
IDH1 mutation status									
Wild type	17 (94.4)	6 (75.0)	0.215	61 (98.4)	4 (21.1)	**<0.001**	290 (94.2)	21 (37.5)	**<0.001**
Mutant	1 (5.6)	2 (25.0)		1 (1.6)	15 (78.9)		18 (5.8)	35 (62.5)	
Sample type									
Primary	15 (83.3)	8 (100)	0.529	49 (79.0)	17 (89.5)	0.501	308 (100)	56 (100)	–
Recurrence	3 (16.7)	0 (0)		13 (21.0)	2 (10.5)		0 (0)	0 (0)	

Bolding indicates that the p‐value is statistically significant.

Abbreviations: ANN, artificial neural network; IDH1, isocitrate dehydrogenase 1; MGMT, O6‐methylguanine DNA methyltransferase; NR, nonresponder; PD1, programmed cell death protein 1; PD‐L1, programmed death‐ligand 1; R, responder; TCGA, The Cancer Genome Atlas.

### 3.2 Construction and evaluation of an ANN model for predicting responders and survival of glioblastoma patients

**FIGURE 2:**
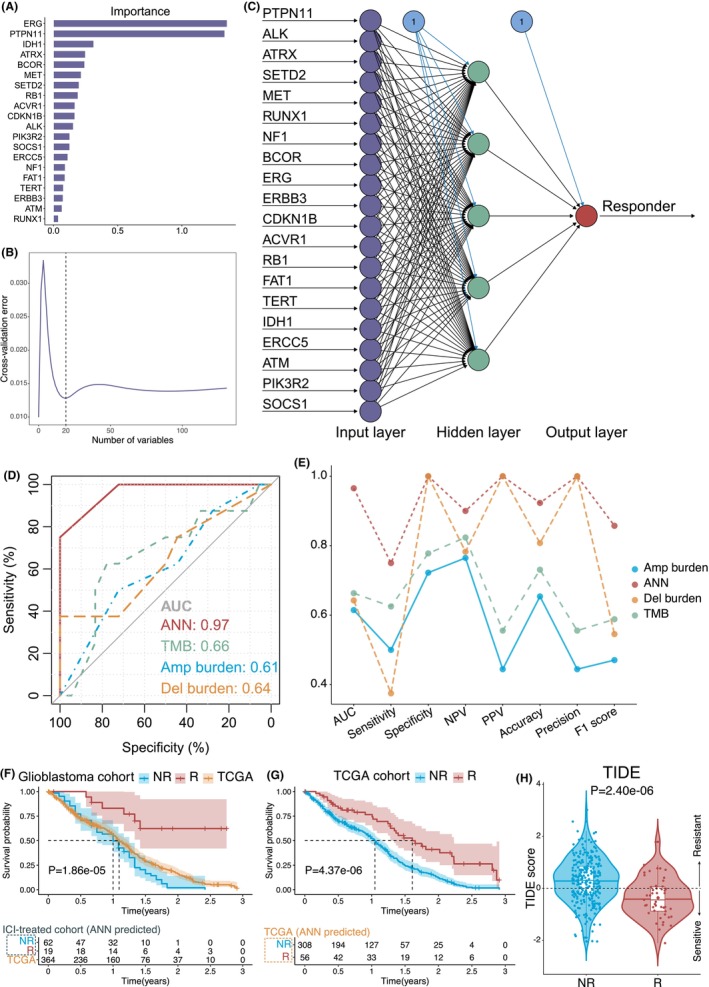
Construction of an ANN model for predicting responders to ICI therapy and survival of glioblastoma patients. (A) The importance plot of the top 20 most critical responder‐relevant variables determined by RF analysis. (B) The 10‐fold cross‐validation plot identified 20 as the optimal number of variables with the lowest cross‐validation error. (C) ANN model. Purple nodes represent input variables, green nodes represent hidden neurons, red nodes represent output neurons, and blue nodes represent the intercepts of each neuron. (D) ROC curves for evaluating the predictive abilities of the ANN model, TMB, and the burdens of copy number amplifications and deletions. (E) Line plots showing comparisons of the AUC, sensitivity, specificity, NPV, PPV, accuracy, precision, and F1 score outcomes among the four predictors. (F) Kaplan–Meier survival analysis of the OS rate among ANN‐predicted responders and nonresponders in the ICI‐treated cohort, as well as TCGA patients. (G) Kaplan–Meier survival analysis of the OS rate between ANN‐predicted responders and nonresponders in the TCGA cohort. (H) Comparison of TIDE scores between ANN‐predicted responders and nonresponders in the TCGA cohort. A TIDE score <0 was considered sensitive to immunotherapy, and a TIDE score >0 was considered resistant to immunotherapy.

RF analysis selected the top 20 genes as the most critical responder‐relevant features with the lowest cross‐validation error (Figure 2A,B). Then, an ANN model with 20 input variables (mutation status), five hidden neurons, and one output neuron was developed in the training set (Figure 2C); the weights of the nodes are displayed in Table S2. ROC analysis and the DeLong test demonstrated that compared with TMB and CNA burdens, the ANN model had the best performance in discriminating responders and nonresponders, with an AUC of 0.97, sensitivity of 75%, specificity of 100%, NPV of 90%, PPV of 100%, accuracy of 92%, precision of 100%, and F1 score of 86% (Figure 2D,E).

Then, the ANN model was further applied to all ICI‐treated and TCGA patients. The ICI‐treated patients were divided into 62 nonresponders and 19 responders. Survival analysis indicated that ANN‐predicted responders had significantly better OS rates than both nonresponders and TCGA patients (Figure 2F), different from Figure 1A. We also classified TCGA patients without immunotherapy into two groups predicted by the ANN model, and survival analysis demonstrated better OS rates for responders (*p* = 4.37e‐06) (Figure 2G). Finally, the TIDE score was calculated for each TCGA patient. ANN‐predicted responders had significantly lower scores than nonresponders, suggesting that the ANN model based on mutation profiles was consistent with the TIDE algorithm in predicting the immunotherapy response (Figure 2H).

## 4 DISCUSSION

Our study has addressed several fundamentally important questions in glioblastoma immunotherapy. Because most glioblastoma patients did not benefit from ICI treatment, no significant difference was observed in OS between the two cohorts of glioblastoma patients with and without immunotherapy. Hence, we used two groups of ICI‐treated glioblastoma patients with large differences in survival benefits to define nonresponders and responders. Then, an ANN model, composed of the mutation status of 20 genes, was developed to predict the immunotherapy response and prognostic benefits of glioblastoma patients. The predictive performance of the ANN model in glioblastoma was much better than that of other widely used predictors, such as TMB. Finally, when applied in clinical practice, the ANN model could guide oncologists in selecting potential responders more accurately for the preferential use of ICIs. Large‐scale, multicenter prospective studies are warranted in the future.

ICI is a very important therapeutic approach in immunotherapy. It blocks tumor‐derived checkpoint‐like molecules, enhances tumor recognition and destruction by endogenous T cells, removes the inhibition of the immune response against tumors, and then inhibits tumor growth. Anti‐PD‐1 is one of the most widely studied ICIs and has achieved satisfactory therapeutic effects in non‐small cell lung cancer, melanoma, renal cancer, colorectal cancer, and gastric cancer, etc.^21^, ^22^, ^23^, ^24^ However, the results of clinical trials conducted in glioblastoma patients are not satisfactory.^20^

Previous studies have suggested that the expression level of PD‐L1 in tumors is related to the therapeutic effect of anti‐PD‐1/PD‐L1. However, even though PD‐L1 is highly expressed in glioblastoma, its predictive value for immunotherapy of GBM is still unclear.^25^, ^26^ It can be inferred that in addition to PD‐1/PD‐L1, there are other pathways that may play an important role in this process, and clarifying the interaction between these pathways will further help clinicians to find candidate biomarkers of anti‐PD‐1/PD‐L1 therapy.

There are several methods to predict ICI response in solid tumors, such as using DNA methylation status,^27^ FDG‐PET/CT,^28^ and Mismatch repair deficiency.^29^ TMB, on the contrary, is one of the more widely used methods. TMB has been shown to have a significant positive correlation with immune‐related adverse events during immune checkpoint inhibitor therapy in a variety of solid tumors.^30^ The reliability of TMB in predicting RESPONSE in ICI‐treated patients has been investigated in colorectal cancer,^31^ breast cancer,^32^ and non‐small cell lung cancer,^33^ among other cancer types. However, as a tumor with relatively small TMB and low incidence, the reliability of TMB in glioblastoma to predict ICI response has not been confirmed. A strong predictive effect of TMB on ICI response was also not found in this study for the time being.

The IDH1/2 mutation plays an important role in glioma. Kohanbash et al.^34^ demonstrated that the occurrence of IDH1/2 mutation may inhibit the aggregation of effector T cells in tumor patients, while the use of IDH inhibitor can effectively increase the infiltration of these cells in tumors, suggesting that IDH1/2 may be a biomarker of immunotherapy. As for gliomas, studies have indicated a D‐2‐HG‐related epigenetic suppression of PD‐1 and PD‐L1 by DNA methylation in IDH‐mutant gliomas.^35^ IDH1 mutations impact the immune landscape of gliomas by affecting immune infiltrations and manipulating checkpoint ligand PD‐L1 expression.^36^ Because of these mechanisms, clinical trials combining PD‐1/PD‐L1 inhibitors and IDH inhibitors are currently underway (NCT04056910). EGFRvIII is also a mutation with high frequency in glioblastoma. Therefore, some researchers believe that this mutation may also be a potential marker of immunotherapy, but further studies are needed to confirm this hypothesis.^37^, ^38^ Zhao et al.^39^ found that the mutation rate of PTEN among nonresponders was higher than that of responders in a study of 66 patients with recurrent glioblastoma who had received PD‐1 blockade. Studies in melanoma also found that PTEN deficiency in tumor cells increased the expression of immunosuppressive cytokines, leading to reduced T cell infiltration, inhibited autophagy, and decreased T cell‐mediated cell death in tumors,^40^ suggesting that the mutation status of PTEN may also become an important biomarker.

In addition, there are other possible immunotherapeutic biomarkers, such as MMR protein deficiency and POLE mutations found by Bouffet et al.^41^ and Johanns et al.^42^ in case reports. Although TILs always show exhausted status in glioblastoma, their existence is still the basis for the function of immunotherapy, so they can also be used as markers of immunotherapy.^38^ Moreover, NK cells have also been proven to be activated by the interaction between PD‐1/PD‐L1. Therefore, PD‐1/PD‐L1 blockade may have a therapeutic effect by activating NK cells, thus making NK cells a marker of immunotherapy.^43^ Although biomarkers of immunotherapy in glioblastoma have been widely studied, there are few biomarkers that can predict the therapeutic efficacy of glioblastoma treated with PD‐1/PD‐L1 blockade. This suggests that we should not look for a single marker; rather, we should consider the influence of various factors comprehensively, so as to build a better prediction model, and the ANN model may provide an approach for this purpose.

ANN is a kind of machine learning that can be used to recognize complex patterns in data. It is currently used in research and clinical practice in a variety of tumors, the most widely used of which is prostate cancer.^44^ This approach plays an important role in the diagnosis, grading, and prognosis of prostate cancer. Moreover, Gregory R. Hart and Mojtaba Sepandi have applied this tool to analyze the clinical data of patients with lung cancer and breast cancer, including imaging data and personal health information, respectively, to predict the occurrence of tumors.^45^, ^46^ In addition, ANN is also used in the analysis of peripheral blood markers and clinicopathological parameters in gastric cancer, so as to predict the long‐term prognosis of patients.^47^ This technique has also been used in the study of glioma, mainly focusing on radiological diagnosis and pathological grading.^10^, ^48^ Recently, this tool has also been applied to the prediction of therapeutic effect, such as the surgical resectability of glioblastoma.^49^ Considering the poor effect of immunotherapy in glioblastoma and the absence of ideal biomarkers, the application of this method to predict the effect of immunotherapy may be able to better screen out glioblastoma patients who can benefit from PD‐L1/PD‐L1 blockade, thus allowing the possibility of immunotherapy.

Although the ANN model is an excellent machine learning model, there is still a certain risk of prediction errors. Therefore, we should be more cautious when applying the ANN model to guide the clinical treatment of glioblastoma patients. In addition, due to the lack of transcriptomic data from ICI‐treated patients, we could not compare the predictive ability between ANN and immune cell infiltration and expressions of checkpoint molecules.

## 5 CONCLUSION

In this study, we defined two groups of ICI‐treated glioblastoma patients with large differences in survival benefits as nonresponders and responders. By performing RF analysis, we selected the most critical responder‐relevant features and developed a 20‐gene panel by ANN. This panel could be a promising biomarker for predicting immunotherapy response of glioblastoma patients treated by ICIs and may help oncologist better identify patients who might benefit from ICIs treatment.

## AUTHOR CONTRIBUTIONS

**Yaning Wang:** Formal analysis (equal); writing – original draft (equal); writing – review and editing (equal). **Zihao Wang:** Conceptualization (equal); formal analysis (equal); writing – original draft (equal). **Xiaopeng Guo:** Conceptualization (equal). **Yaning Cao:** Writing – original draft (equal); writing – review and editing (equal). **Hao Xing:** Data curation (equal). **Yuekun Wang:** Investigation (equal); validation (equal); visualization (equal). **Bing Xing:** Resources (equal). **Yu Wang:** Conceptualization (equal); funding acquisition (equal). **Yong Yao:** Conceptualization (equal); supervision (equal). **Wenbin Ma:** Funding acquisition (equal); supervision (equal).

## CONFLICT OF INTEREST STATEMENT

The authors have no conflicts of interest to declare.

## ETHICS STATEMENT

The data for our study were obtained from published databases, so ethical approval statement is not applicable.

## Supporting information


Table S1.

Table S2.

## Data Availability

The somatic mutational and clinical data of the ICI‐treated cohort were downloaded from cBioPortal (https://www.cbioportal.org/study/summary?id=tmb_mskcc_2018) from a large‐scale clinical trial.^12^ Corresponding clinical information of The Cancer Genome Atlas (TCGA) glioblastoma cohort with no ICI treatment were downloaded from GDC data ortal (https://por tal.gdc.cancer.gov/).
